# Introduction of Typhoid Conjugate Vaccines in Africa and Asia

**DOI:** 10.1093/cid/ciy878

**Published:** 2019-02-15

**Authors:** Kathleen M Neuzil, Andrew J Pollard, Anthony A Marfin

**Affiliations:** 1 Center for Vaccine Development and Global Health, University of Maryland School of Medicine, Baltimore; 2 Oxford Vaccine Group, Department of Paediatrics, University of Oxford, United Kingdom; 3 PATH, Seattle, Washington

**Keywords:** typhoid, vaccine, TyVAC, Africa, Asia

## Abstract

Typhoid fever continues to be a major public health concern, particularly in many low- and middle-income countries. The current threats of increasing antimicrobial resistance, urbanization, and climate change elevate the urgency for better prevention and control efforts for typhoid fever. In 2017, the results of ground-breaking research on typhoid conjugate vaccines (TCVs), the World Health Organization prequalification of a TCV, and global policy and financing decisions have set the stage for the introduction of TCVs into routine immunization programs in endemic countries. Country-level decision-making and program planning are critical for local uptake and sustainability.

Typhoid, a serious and sometimes fatal disease caused by the bacterium *Salmonella enterica* serovar Typhi (*S*. Typhi), is spread through contaminated food and water and poor sanitation. Although typhoid has been largely eliminated in industrialized countries, it continues to be a substantial public health problem in many low- and middle-income countries. Globally, there are nearly 12 million cases and more than 128 000 deaths due to typhoid each year [1]. The burden is likely underestimated due to lack of comprehensive surveillance data, poor sensitivity of available diagnostics, and their limited deployment. Current trends in antibiotic resistance, urbanization, and climate change may increase the risk for typhoid. While improved water quality, sanitation, and hygiene (WASH) are the major ways to break the typhoid transmission cycle, until these investments are made in all countries, vaccination is an important and effective option to prevent typhoid.

To reduce the burden of *S*. Typhi, the Bill & Melinda Gates Foundation funded the Typhoid Vaccine Acceleration Consortium (TyVAC) in late 2016. TyVAC is led by the Center for Vaccine Development and Global Health at the University of Maryland School of Medicine, the Oxford Vaccine Group at the University of Oxford, and PATH. TyVAC uses a multidisciplinary, iterative approach to accelerate the introduction of typhoid conjugate vaccines (TCVs) into low- and middle-resource countries, particularly those eligible for support from Gavi, the Vaccine Alliance.

TyVAC is working closely with the World Health Organization (WHO), Gavi, and other local and global stakeholders to design and execute a strategy based on prior successful new vaccine introductions. Core activities are customized to address challenges posed by typhoid fever and demonstrate TCV introduction for optimal impact.

## TYPHOID VACCINES: CURRENT STATUS

In 2018, typhoid continues to be a public health threat. Children and adolescents in Asia and sub-Saharan Africa are disproportionately impacted by typhoid, with those living in poor communities at the greatest risk. In addition to the disease burden, global outbreaks and growing antimicrobial resistance further highlight the need for prevention. In and around Harare, Zimbabwe, for example, an outbreak has been ongoing since October 2017, with 200 new cases reported in the first few weeks of 2018 [2]. In Pakistan, the first large outbreak of third-generation cephalosporin-resistant *S*. Typhi occurred in 2017, greatly complicating treatment options [3].

Prior to 2017, 2 typhoid vaccines were available: the oral, live attenuated Ty21a vaccine licensed for children aged ≥6 years, and an injectable Vi capsular polysaccharide vaccine licensed for children aged ≥2 years [4]. Only the latter is prequalified by WHO. These vaccines are underutilized in high-burden countries despite typhoid’s substantial and detrimental impact and WHO recommendation for their use. It has been difficult to incorporate these vaccines into routine immunization programs in low-resource countries because neither is approved for children aged <2 years or subsidized by Gavi.

## ADVANTAGES OF TCVS

New TCVs have the potential to overcome challenges that have impeded the uptake of earlier vaccines. TCVs have longer-lasting protection, require fewer doses, and are suitable from infancy, allowing delivery through routine childhood immunization programs. Expanded use of TCVs through routine immunization has the potential to reduce the need for antibiotics, slow further emergence of drug-resistant typhoid strains, and save lives.

Typbar-TCV, manufactured by Bharat Biotech International Limited, is safe, well tolerated, and induces a robust and long-lasting response across age groups for longer periods of time than a currently prequalified polysaccharide typhoid vaccine [4]. The high immunogenicity of Typbar-TCV makes it an ideal candidate in typhoid-endemic countries, especially for children aged <2 years, a group particularly vulnerable to typhoid fever. Researchers at Oxford University conducted the first clinical trial to assess the efficacy of Typbar-TCV using a controlled human infection model. Results showed that the vaccine halved the total number of typhoid cases and had an efficacy of 87.1% when endpoints of fever >38°C followed by a positive blood culture were used [5]. Interestingly, this estimate was aligned with an analysis of serological evidence of protection in an immunogenicity trial in India [6]. These data were essential supporting evidence for global policy decisions.

Successful and sustainable vaccine introduction requires supporting evidence, government backing, financing, WHO prequalification, country readiness/willingness, political will, and local endorsement. Within a few short months in 2017, Typbar-TCV was recommended by WHO’s Strategic Advisory Group of Experts (SAGE) on Immunization, supported by Gavi, and prequalified by WHO. In October, SAGE recommended a single-dose TCV for infants and children aged >6 months in typhoid-endemic countries; introduction prioritized in countries with the highest burden of disease or a high burden of antimicrobial-resistant *S*. Typhi; a catch-up vaccination strategy, when feasible, for children up to age 15 years, depending on local epidemiology; and typhoid vaccination in response to confirmed outbreaks of typhoid fever and considered in humanitarian emergencies [7, 8]. In November, Gavi announced $85 million to support the introduction of TCVs [9]. This funding opens the way for low-income, high-burden, Gavi-eligible countries to affordably introduce safe and effective typhoid vaccines. Country applications will be accepted in the second half of 2018 for introductions in 2019 and 2020. In December, WHO prequalified the first TCV, that is, Typbar-TCV [10, 11]. Prequalification means the TCV has the WHO stamp of approval and can be purchased by countries through United Nations procurement agencies, including WHO, the United Nations Children’s Fund, and Gavi. While Typbar-TCV is the first prequalified TCV, many more TCVs are in development [unpublished data]. The availability of additional TCVs will ensure global supply and a competitive marketplace.

Multiple delivery strategies may be used to operationalize the WHO recommendations. Considerations and challenges for a delivery strategy include risk-based vs universal coverage under routine immunization, catch-up, and use in endemic areas and/or during outbreaks. It is challenging to target high-risk individuals because the risk may be based on the setting or population and may vary over time. In addition, it can be hard to identify high-risk individuals in advance due to population expansion and movement. Incorporating TCV into the routine immunization schedule allows protection for all children prior to exposure to typhoid, which is being increasingly recognized in young children [8, 12].

There are challenges related to routine immunization, such as vaccine supply, vaccine cost, and the increasing number of vaccines administered at 9 to 15 months. WHO SAGE called for further studies to understand tolerability and immunogenicity of Typbar-TCV coadministered with other Expanded Programme on Immunization–recommended vaccines (the Expanded Program on Immunization is a World Health Organization program to expand immunization programs and make vaccines more accessible). Understanding the duration of immunity through ongoing studies is critical to inform strategies.

Vaccine introduction and the associated policy-making processes are complex, often fragmented, and involve many groups across government. Lessons learned from new vaccine adoption and introduction are that global action drives country decisions through WHO recommendations and affordable vaccines, support from Gavi for vaccine introduction is extremely important, and processes used by countries that successfully introduce new vaccines are largely similar [13, 14]. Introduction requires a commitment by the country’s national immunization program to determine the feasibility and acceptability of introduction to generate robust financial support for a sustainable program.

Demonstration of the impact of new vaccines in different endemic populations is critical. Prior to the TyVAC project, there were no field efficacy studies for Typbar-TCV, and challenge studies may not predict field effectiveness. As a major component of TyVAC, we are implementing vaccine evaluations of Typbar-TCV to provide data to support the introduction of the vaccine in those regions suffering a substantial burden of disease. Our proposed studies have been designed to be complementary to each other and to other efforts (Table 1) [unpublished data]. In addition to efficacy of culture-confirmed typhoid fever, other outcomes include safety, immunogenicity, costs, and effects on other important public health outcomes such as antibiotic usage. We will conduct individually randomized, controlled trials in Nepal and Malawi among children beginning at age 9 months, with a 1:1 randomization of TCV or the control vaccine, a serogroup A meningococcal conjugate vaccine. In Bangladesh, we will conduct a controlled cluster randomized trial. In this design, rather than randomizing individuals to TCV or the live, attenuated Japanese encephalitis control vaccine, we will randomly allocate groups (or clusters) of individuals to treatment arms [15]. Our primary reason for using this design is to determine if the vaccine can prevent typhoid by protecting the vaccinated individuals and also by reducing transmission of typhoid to those in the community who have not been vaccinated (ie, indirect protection). The groundwork for the TyVAC sites has been provided by the Strategic Typhoid Alliance across Africa and Asia [16], which was conducted at the 3 trial sites. The large populations under surveillance for blood culture–confirmed typhoid fever in the 3 countries informed our study design and sample sizes. In Malawi and Nepal, passive surveillance for febrile illness will be conducted at community-based health clinics and local hospital for all vaccinated children. In Bangladesh, the surveillance will cover all vaccinated children and unvaccinated cluster residents.

**Table 1.** Summary of Typhoid Vaccine Acceleration Consortium Impact Studies

Site	Nepal (Kathmandu)	Malawi (Blantyre)	Bangladesh (Dhaka)
Trial design	Individually randomized, controlled trial	Individually randomized, controlled trial	Cluster randomized, controlled trial
Investigational vaccine	Single-dose Vi-TCV Licensed trade name: Typbar-TCV, Bharat-Biotech		
Comparator (control) vaccine	Serogroup A meningococcal conjugate vaccine	Serogroup A meningococcal conjugate vaccine	Live attenuated Japanese encephalitis
Sample size targets	20 000	28 000	43 350 within 150 clusters
Participant age	9 months to <16 years	9 months through <13 years	9 months to <16 years
Start date	November 2017	February 2018	March 2018
Primary outcome	Determine efficacy and rate reduction of Vi-TCV in preventing blood culture–confirmed symptomatic infection by Salmonella enterica serovar Typhi		
Study duration, months	30	36	30
Safety follow-up	• Immediate reactions • SAEs • AEs 7 days post-vaccination	• Immediate reactions and SAEs in all participants • Subset: local and systemic solicited and unsolicited reactions for 7 days post-vaccination • Subset: all AEs for 28 days post-vaccination	• Immediate reactions • SAEs • AEs 7 days post-vaccination
Immunogenicity data (subset)	Anti-Vi antibodies on day 0, 1 month (day 28), day 545, and day 730	Anti-Vi antibodies on day 0, 1 month (day 28), and day 730	Anti-Vi antibodies on day 0, 1 month (day 28), and day 730
Co-administration with other vaccines (measles-rubella)	Not applicable	Measles and rubella antibody response in 200 children aged 9–11 months	Not applicable
Surveillance	Passive surveillance for febrile illness in vaccinated children at community-based health clinics and local hospital	Passive surveillance for febrile illness in vaccinated children at community-based health clinics and local hospital	Passive surveillance for febrile illness in all cluster residents at community-based health clinics and local hospital
Follow-up duration	2-year follow-up post-vaccination for each participant	Minimum of 2 years follow-up post-vaccination for each participant or until the number of verified cases is reached	2-year follow-up post vaccination for each participant

Abbreviation: AE, adverse event; SAE, serious adverse event; Vi-TCV, Vi polysaccharide-tetanus toxoid conjugate vaccine.

From the publication of pivotal typhoid research to local and national efforts against typhoid outbreaks, 2017 and 2018 have been exciting years for efforts to take on typhoid. The rise of drug-resistant typhoid combined with global trends of urbanization and climate change, which can increase risk factors for the spread of typhoid, have raised the urgency of prevention. Thanks to the hard work of many global partners, we are raising awareness of the burden of typhoid and accelerating access to TCVs. This year, we continue to work with countries to help them decide if, when, and how to introduce TCVs along with WASH interventions as part of a comprehensive approach to typhoid prevention and control. Additional information on TyVAC and the initiative to take on typhoid is available on our website [17].

## Notes

***Acknowledgments.*** The authors acknowledge Leslie Jamka for her contributions to writing and reviewing this commentary.

***Financial support.*** This work was supported by the Bill & Melinda Gates Foundation (grantOPP1151153).

***Supplement Sponsorship.*** This supplement was sponsored by Coalition against Typhoid, Sabin Vaccine Institute, Bill & Melinda Gates Foundation.

***Potential conflicts of interest.*** All authors: No reported conflicts. All authors have submitted the ICMJE Form for Disclosure of Potential Conflicts of Interest. Conflicts that the editors consider relevant to the content of the manuscript have been disclosed. A. J. P. is a member of the WHO’S SAGE.
